# Does Unidirectional Block Exist after a Radiofrequency Line Creation? Insights from Ultra-High-Density Mapping (The UNIBLOCK Study)

**DOI:** 10.3390/jcm10112512

**Published:** 2021-06-06

**Authors:** Sok-Sithikun Bun, Antoine Da Costa, Jean-Baptiste Guichard, Ziad Khoueiry, Fabien Squara, Didier Scarlatti, Philippe Taghji, Pamela Moceri, Emile Ferrari

**Affiliations:** 1 Cardiology Department, Pasteur University Hospital, 06000 Nice, France; squara.f@chu-nice.fr (F.S.); scarlatti.d@chu-nice.fr (D.S.); moceri.p@chu-nice.fr (P.M.); ferrari.e@chu-nice.fr (E.F.); 2 Cardiology Department, Nord University Hospital, 42055 Saint-Etienne, France; dakosta@orange.fr (A.D.C.); jeanbaptisite.guichard@gmail.com (J.-B.G.); 3 Cardiology Unit, Perpignan Private Hospital, 66000 Perpignan, France; ziad.khoueiry@gmail.com; 4 Cardiology Department, Timone University Hospital, 13385 Marseille, France; philippetaghji@hotmail.com

**Keywords:** unidirectional block, radiofrequency line, ultra-high-density mapping

## Abstract

Background: Whether unidirectional conduction block (UB) can be observed after creation of a radiofrequency (RF) line is still debated. Previous studies reported a prevalence of 9 to 33% of UB, but the assessment was performed using a point-by-point recording across the line. Ultra-high-density (UHD) system may bring some new insights on the exact prevalence of UB. Purpose: A prospective study was conducted to assess the prevalence of UB and bidirectional block (BB) using UHD system after RF line creation. Methods: Patients referred for atrial RF ablation procedure were included in this multicenter prospective study. UHD maps were performed by pacing both sides of the created line. Results: A total of 80 maps were created in 40 patients (67 ± 12 years, 70% male) by pacing (mean cycle length 600 ± 57 ms) from both sides of the cavotricuspid isthmus line. After a 47 ± 17 min waiting time after the last RF application, UHD maps (mean number of 4842 ± 5010 electrograms, acquired during 6 ± 5 min) showed that BB was unambiguously confirmed on all of them. UB was not observed in any map. After a mean follow-up of 12 ± 4 months, 6 (14%) patients experienced an arrhythmia recurrence. Conclusion: After creation of an RF line, no case of UB was observed using UHD mapping, suggesting that the presence of a conduction block along a RF line is always associated with a block in the opposite direction.

## 1. Introduction

Exclusive unidirectional electrical conduction can be observed in clinical situations such as complete atrioventricular block with intact retrograde conduction, allowing pacemaker-mediated tachycardias. Exclusive unidirectional conduction may also be observed through accessory pathways with concealed retrograde conduction without antegrade pre-excitation [1].

In the field of macroreentrant atrial arrhythmias, validation of bidirectional block (BB) across the radiofrequency (RF) line is critical to ensure efficacy of ablation. However, UB has been reported in previous studies using standard mapping technology, and may be associated with wrongly classified failed ablation procedure or prolonged RF delivery with potential harmful consequences. In previous studies, the conduction block was assessed by pacing on one side of the line, and recording on the other side of the line, with a single bipolar catheter, i.e., at a unique recording point on the other side of the line [2]. We conducted a multicenter prospective study to assess the prevalence of bidirectional block (BB) and UB using an ultra-high-density (UHD) system after the RF line in human atria.

## 2. Materials and Methods

### 2.1. Patient Population

From January 2020 to January 2021, patients referred for RF atrial arrhythmia ablation in the three participating French centers (Nice University Hospital, Saint-Etienne University Hospital, Perpignan Private Hospital) underwent UHD mapping. Patients with macroreentrant mechanisms involving cavotricuspid isthmus (CTI) were included in this single-arm prospective study.

All patients had symptomatic atrial arrhythmias. Exclusion criteria were hyperthyroidism, LA thrombus, decompensated heart failure, stroke, myocardial infarction, or gastrointestinal bleeding within 4 weeks prior to the intervention, and life-expectancy < 6 months. According to institutional guidelines, all patients gave written informed consent for the study and procedures. The study was approved by the institutional review board.

### 2.2. Atrial Arrhythmia Ablation Procedures

Procedures were performed with vitamin K antagonist continuation with a target International Normalized Ratio of 2.0–3.0, or uninterrupted direct oral anticoagulant (dabigatran, rivaroxaban, or apixaban given the evening before the procedure if LA arrhythmia considered), under conscious sedation or general anesthesia in a fasting state. All catheters were advanced via the femoral vein after ultrasound-guided venous puncture [3]. A 6F steerable catheter was positioned in the coronary sinus (CS), and served as a reference (Irvine Bio Inc., St. Jude Medical Inc., St. Paul, MN, USA; or 12-pole catheter, Woven^®^, Boston Scientific). A 3.5 mm open-irrigated contact-force sensing tip (Tacticath Quartz^®^, Abbott Medical, IL, USA), or impedance-based catheter (Intella Nav Mifi^®^, Boston Scientific, Natick, MA, USA) was then advanced for CTI ablation. All procedures were performed by three experienced electrophysiologists.

CTI ablation was then performed starting from the ventricular side towards the caval side, targeting a force-time integral (FTI) > 400 gs in a point-by-point manner, as previously described [4]. In case of CTI-dependent flutter, procedure endpoint was arrhythmia interruption with sinus rhythm restoration, and demonstration of a conduction block across the line using electroanatomic activation mapping with UHD system (Rhythmia^®^, Boston Scientific, Natick, MA, USA) after classical electrophysiological criteria of CTI conduction block have been presented: an activation sequence suggestive of clockwise CTI block at the low lateral right atrium (RA) when pacing at the CS ostium, and the presence of a corridor of separated double potentials all along the ablation line [5]. Power was set at 35 to 45 W, with a flush rate of 17–25 mL/min. 

### 2.3. Conduction Block Assessment

After completion of each RF line and a waiting period of at least 30 min, a UHD activation map was performed to evaluate if endocardial conduction block was present and, if present, if there was UB or BB. Differential site pacing was performed prior to UHD activation mapping. Electroanatomic maps were then performed while pacing on one side of the line (cycle length 500, 600 or 700 ms), and another map was consecutively performed when pacing from the other side of the line, using either the decapolar catheter or the ablation catheter. The first activation map was performed by pacing on the proximal CS, and the second map was performed when pacing with the ablation catheter on the low lateral RA. Detailed electroanatomic mapping of the RA was performed using the bidirectional flexion with the basket (Orion^®^, Boston Scientific, Natick, MA, USA) in variable degrees of deployment (diameter ranging 3–22 mm) [6]. A detailed analysis of the completion of the line was performed and aimed to visualize double potentials using the virtual catheter all along the ablation line.

The number of UB and bidirectional blocks (BB) were analyzed for each RF line.

### 2.4. Statistical Analysis

Considering that UB prevalence using conventional mapping was 9–33%, based on previous studies [7], and expecting that UB prevalence using UHD mapping was 9% or less, a sample size of 30 patients was calculated to achieve a power of 90% and to detect a significant difference with a two-sided 0.05 alpha level (epiR package 0.9-96, Melbourne, Australia). The statistical analysis was made with Excel (San Diego, CA, USA). Categorical variables are described as numbers and percentages. Continuous variables are described as mean ± SD for variables with normal distributions or as median with range for variables not normally distributed.

## 3. Results

**Figure 1:**
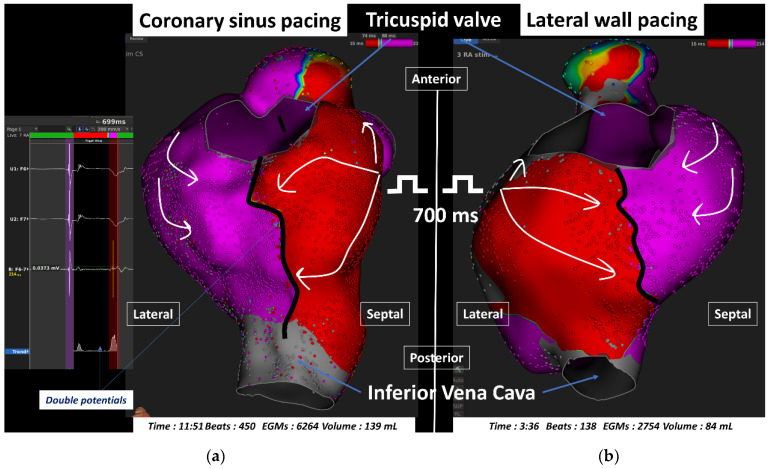
Ultra-high density electroanatomic right atrial activation maps performed sequentially (in the same patient, 30 min) after radiofrequency cavotricuspid isthmus line creation, in left anterior oblique projection at 45°, and tilted in an inferior view. The left map (**a**) represents an activation map when pacing (700 ms) at the proximal coronary sinus, while the right map (**b**) has been performed when pacing on the low lateral right atrium with the ablation catheter. The line of block is represented by a bold black line. Electrograms show widely separated double atrial potentials with very late second atrial potential along the entire endocardial ablation line during coronary sinus pacing.

A total of 80 maps (40 pairs of maps) were created in 40 patients (67 ± 12 years, male 70%) by pacing (mean cycle length 600 ± 57 ms) from both sides of the CTI line (Figure 1). Three patients underwent additional RF applications on the CTI, because of conduction recovery across the line within the 30 min waiting time. UHD maps checking for conduction block were performed after 30 min of extra-time after the last RF pulse. A total of 12 patients (30%) underwent concomitant LA lesions in addition with CTI ablation within the same procedure: PV isolation (*n* = 8), roof line (*n* = 2), complex fractionated atrial electrogram ablation (*n* = 3), and localized LA ablation for microreentrant circuits (*n* = 2 on the ridge, and the LA appendage base). The clinical and procedural characteristics are reported in Table 1. After a 47 ± 17 min waiting time after the last RF application, UHD maps (mean number of 4842 ± 5010 electrograms, acquired during 6.8 ± 5 min; 438 ± 510 beats) showed that BB was unambiguously confirmed on all of them. UB (or pseudo UB) was not observed in any map. The mean RA volume was 113 ± 74 mL. 

After a mean follow-up of 12 ± 4 months, four (10%) patients experienced arrhythmia recurrence after the 3-month blanking period: paroxysmal atrial fibrillation (AF) for two of them, paroxysmal LA tachycardia, and one patient presented a CTI-dependent flutter recurrence. All the patients had symptoms controlled under anti-arrhythmic drug therapy, while the fourth patient was proposed a successful repeat CTI ablation procedure.

## 4. Discussion

Our study specifically used a UHD activation mapping system to assess conduction block (UB or BB) after RF line ablation. Confirmation of complete BB is mandatory after RF line creation, to prevent any atrial tachycardia recurrence involving isthmus tissue residual conduction. Ensuring line completeness is a class I recommendation in the last international guidelines on AF ablation [8].

In previous studies, the conduction block was assessed by pacing on one side of the line, and recording on the other side of the line, with a single bipolar catheter, i.e., at a unique recording point on the other side of the line [2]. This lack of resolution may explain the existence of UB (9 to 33% of the cases) [7].

Our study demonstrates that once an RF line is created at the CTI level, an endocardial conduction block (CW for instance when pacing on the proximal CS at the CTI level) in one direction always implies endocardial conduction block in the opposite direction across the line (CCW block when pacing at the low lateral RA). Apparent UB in three patients out of twelve after CTI ablation (studied with a “basket” catheter) was attributed to the presence of conduction along the posterior part of the inferior vena cava orifice, despite complete CTI BB [9]. This initial study emphasized the limitations of BB assessment with non-UHD mapping systems, which still are the rule in the vast majority of electrophysiology laboratories worldwide. Our study was designed to use UHD systems (instead of conventional non-UHD point-by-point confirmation) to evaluate the type of conduction block (UB or BB) after RF line creation. It is well established that multielectrode mapping was superior to point-by-point for accurate diagnosis of arrhythmia mechanism [10,11]. Currently, UHD systems have proven to be highly efficient with automatic annotation for confirmation of conduction block along a line or for highlighting existing gaps [12,13,14]. In the modern era, widespread use of contact-force catheters also contributes to facilitate and achieve complete conduction block across a RF line, and therefore assessment of BB [4]. Mini-electrode technology embedded within the ablation catheter tip may also be useful for conduction block assessment [15].

**Figure 2:**
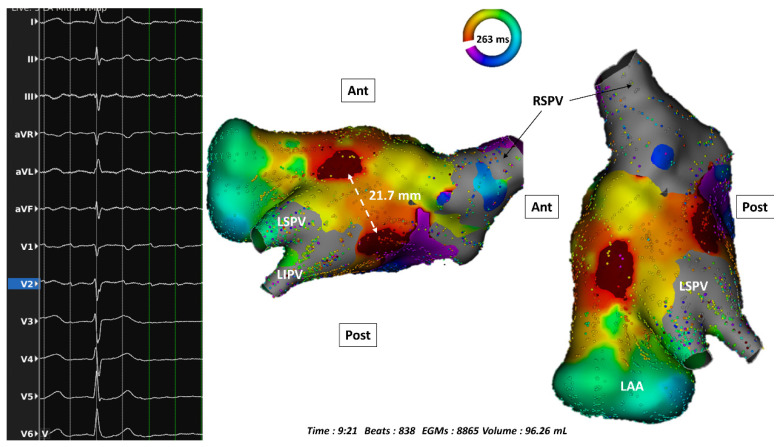
Ultra-high density electroanatomic activation map (during macroreentrant tachycardia) showing a rare case of endo-epicardial breakthrough at the level of a roof line created with radiofrequency delivery. LAA: left atrial appendage; RSPV: right superior pulmonary vein.

However, in rare cases of suspected CTI-dependent flutter which cannot be terminated by ablation despite the presence of a confirmed BB by UHD mapping, alternative diagnosis of intra-isthmus reentry should be ruled out [16]. Very recently, epicardial to endocardial breakthrough at the CTI level has been found in only 4% of the cases, using either conventional mapping (5 patients) [17], or a UHD system [18]. Concerning posterolateral MI ablation, physicians may also be cautious about rare exceptions which are the endo-epicardial breakthrough circuits propagating through the Marshall bundle epicardial connection for instance [19]. A recent study showed that residual conduction through CS and Marshall tract connections (which could be seen in 51% of the cases after endocardial ablation at the MI level) may be at the origin of apparent UB observation [20]. Another example of endo-epicardial circuit (septo-pulmonary bundle) across a LA roof line with complete endocardial BB is provided in Figure 2 [21].

### Limitations

The relatively limited number of patients included in this multicenter prospective study can be explained by the additional cost associated with the use of UHD mapping system compared to the standard approach, which limited inclusions of patients. For this reason, studies using UHD for CTI-dependent flutter usually include small populations [22,23,24]. Furthermore, activation mapping is more time-consuming (mean acquisition time 6.8 ± 5 min per map), in comparison with differential site pacing technique. Although being multicenter, our study included a limited number of patients for the above-mentioned reasons. BB was observed with UHD mapping in most cases after RF line creation, but rare cases of endocardial-epicardial breakthroughs may be seen and have been reported. Further studies may be performed to confirm our results, especially at the PV-antrum level, and/or for LA lines.

## 5. Conclusions

After creation of an RF line, mainly at the CTI, UHD activation maps confirmed that BB was usually the rule. Endocardial UB was not observed in our study. When a conduction block is present across a RF line, the block is bidirectional in the vast majority of the cases. The presence of either clockwise or counterclockwise block is a proof of complete endocardial line of block in the cavotricuspid isthmus, and no additional pacing maneuver is required to confirm the existence of a conduction block in the other direction. While promising, additional studies may still be needed to confirm these findings and further elucidate the role of UHD mapping in the treatment of atrial arrhythmias. 

## Figures and Tables

**Table 1:** Baseline characteristics of study patients (*n* = 40).

Characteristics	Value
Age (years)	67 ± 12
Sex, male (%)	28 men (70)
Body Mass Index (kg/m²)	25.7 ± 3.4
CHA2DS2VASc score	2.66 ± 1.6
Structural heart disease, *n* (%)	18 (45)
Left atrial diameter (mm)	51 ± 12
Left ventricular ejection fraction (%)	54 ±10
Total procedure time (min)	114 ± 52
Fluoroscopy time (min)	10.4 ± 7
Concomitant left atrial lesion ablations, *n* (%)	12 (30)
Follow-up (months)	12 ± 4 months
